# A CRISPR/Cas13-based approach demonstrates biological relevance of vlinc class of long non-coding RNAs in anticancer drug response

**DOI:** 10.1038/s41598-020-58104-5

**Published:** 2020-02-04

**Authors:** Dongyang Xu, Ye Cai, Lu Tang, Xueer Han, Fan Gao, Huifen Cao, Fei Qi, Philipp Kapranov

**Affiliations:** Institute of Genomics, School of Biomedical Sciences, Huaqiao University, 201 Pan-Chinese S & T Building, 668 Jimei Road, Xiamen, 361021 China

**Keywords:** High-throughput screening, Functional genomics, Non-coding RNAs

## Abstract

Long non-coding (lnc) RNAs represent a fascinating class of transcripts that remains highly controversial mainly due to ambiguity surrounding overall biological relevance of these RNAs. Multitude of reverse genetics studies showing functionality of lncRNAs are unfortunately based on assays that are either plagued by non-specific effects and/or cannot unambiguously assign observed phenotypes to the transcript *per se*. Here, we show application of the novel CRISPR/Cas13 RNA knockdown system that has superior specificity compared to other transcript-targeting knockdown methods like RNAi. We applied this method to a novel widespread subclass of nuclear lncRNAs — very long intergenic non-coding (vlinc) RNAs — in a high-throughput phenotypic assay based on survival challenge in response to anticancer drug treatments. We used multiple layers of controls including mismatch control for each targeting gRNA to ensure uncovering true phenotype-transcript relationships. We found evidence supporting importance for cellular survival for up to 60% of the tested protein-coding mRNAs and, importantly, 64% of vlincRNAs. Overall, this study demonstrates utility of CRISPR/Cas13 as a highly sensitive and specific tool for reverse genetics study of both protein-coding genes and lncRNAs. Furthermore, importantly, this approach provides evidence supporting biological significance of the latter transcripts in anticancer drug response.

## Introduction

The lncRNA class of transcripts dominates the transcriptional output of a mammalian genome^1–3^ and as such attracted vast research interest^4,5^. However, despite a large amount of effort dedicated to understanding their functionality, biological relevance of this class remains a subject of a vigorous debate with arguments not only supporting^4–6^ but also challenging^7–9^ the significance of these transcripts. In a large measure, the inability to unambiguously address the relevance of lncRNAs comes from issues associated with the reverse-genetics techniques employed for this task^10^. Broadly speaking, these issues can be subdivided into two categories. First, methods that target DNA and either change genomic sequence or target transcriptional modulators to regulatory elements of the target transcripts often cannot definitively assign a phenotype to the targeted transcript^10^. In fact, a number of recent reports found that phenotypes previously assigned to lncRNAs by genome editing techniques were in fact caused by perturbations of the DNA sequence elements overlapping those transcripts^11–13^. Second, currently-used methods that target transcripts, typically based on RNAi or antisense oligonucleotides (AOs), often have significant non-specific and off-target effects^14–17^. Furthermore, as shown in a recent report by Stojic *et al*., non-specific siRNAs or AOs that are not supposed to target cellular transcripts can nonetheless cause substantial transcriptome changes in a sequence-dependent fashion^18^. These observations suggest that controlling for non-specific effects of siRNAs and AOs is non-trivial and may even not be entirely possible. In fact, a growing number of reports shows that non-specific and off-target effects can indeed lead to erroneous assignment of a phenotype to the targeted transcripts, suggesting that this issue might be widespread^19–21^.

These problems clearly call for development of approaches that can provide unambiguous connection between an observed phenotype and the targeted transcript. Ideally, such approaches should target RNA without any non-specific or off-target effects. The newly-reported CRISPR/Cas13 system appears to represent a significant improvement over the existing RNA-targeting methods^22^. First, it has significantly lower off-target effects compared to RNAi^22^. Second, its activity is severely reduced or abrogated by 1–2 mismatches in the center of a guide (g)RNA, allowing for a mismatch control for each targeting gRNA^22^. The availability of such control that shares most of the sequence with the targeting gRNA would theoretically allow to account for most if not all sequence-specific off-target effects affecting the other knockdown technologies^18^.

However, CRISPR/Cas13 has not been used for functional studies of lncRNAs or mRNAs. Furthermore, it has not been applied in the context of stable cell lines — the original report tested the method only in transient transfection assays^22^. Stable cell lines would be highly desirable in a number of reverse-genetics strategies, such as high-throughput screenings and long-term survival assays, for example. Therefore, in this work, we have explored a possibility of applying CRISPR/Cas13 system for high-throughput phenotypic screens for lncRNAs and mRNAs in a context of a stable expression system. In this design, each cell stably expresses a specific gRNA that upon induction of Cas13 also integrated into the genome can induce knockdown of the target transcript. Thus, each cell is barcoded by a unique gRNA sequence. The effect of the knockdown on cellular viability could then be judged by depletion or enrichment of the specific gRNA barcodes in the cellular population.

We have built in several layers of controls to allow for as accurate measurement of a transcript-related effect on cell survival as currently achievable. To test this system, we have chosen a recently-discovered class of very long intergenic non-coding (vlinc)RNAs that represent nuclear polyA− transcripts of over 50 kb widespread in a mammalian genome^23,24^. These transcripts were implicated in control of cellular senescence^25^ and replication timing^26^, however, for most part, their mechanisms of function and biological significance remain unexplored. The nuclear localization^25,27^, high cell-type specificity^24^ and length make vlincRNAs a highly technically challenging subclass of lncRNAs accounting for the general paucity of functional studies^10^. Using vlincRNAs as an example, we show in this proof-of-principle study that the CRISPR/Cas13 system has significant merit for high-throughput functional assays of lncRNAs. Furthermore, we provide evidence strongly suggesting that majority of the tested vlincRNAs represent functional RNA species.

## Results

### Establishment of a high-throughput screening system based on inducible CRISPR/Cas13

**Figure 1:**
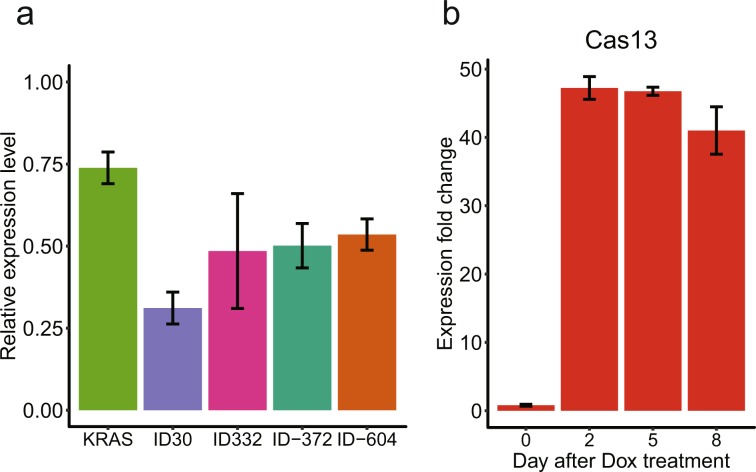
Establishing a stable CRISPR/Cas13 system based on K562 cell line. (**a**) CRISPR/Cas13 can be used to knockdown mRNA and vlincRNAs in K562. Relative expression level (Y-axis) of the corresponding transcripts in cells co-transfected with plasmids expressing Cas13 and the indicated target gRNA vs those co-transfected with Cas13 and the control Gluc gRNA. (**b**) Inducible stable Cas13 expression in TRE-LwCas13a-K562 cells. Expression fold change (Y-axis) in response to +Dox treatments compared to the −Dox control. Error bars indicate the SE of three technical repeats.

As the first step, we tested whether the CRISPR/Cas13 methodology can potentially knockdown vlincRNAs. To accomplish this, we used a co-transfection assay where two plasmids constitutively expressing (1) nuclear-localized Cas13-msfGFP fusion protein (see Materials and Methods for more details) and (2) a specific gRNA are electroporated into K562 chronic myeloid leukemia (CML) cells followed by assessing the depletion of the target transcript after 24 h using RT-qPCR. We used a positive control gRNA against protein-coding mRNA *KRAS* employed in the original study describing the CRISPR/Cas13 system^22^ and a gRNA targeting *Gaussia* luciferase (Gluc) from the same source as the non-specific control. Indeed, we could achieve a statistically-significant reduction in the *KRAS* mRNA in cells transfected with the corresponding gRNA relative to the Gluc gRNA control (p-value < 0.05, Student’s *t*-test, Fig. 1a). As the next step, we tested CRISPR/Cas13 knockdown using gRNAs against 4 vlincRNAs (Supplementary Table 1). Indeed, we could observe a statistically-significant depletion of each of the tested vlincRNAs (p-value < 0.05, Student’s *t*-test, Fig. 1a).

Encouraged by these results, we then generated a stable K562 cell line expressing the same Cas13-msfGFP protein however in a doxycycline (Dox)-inducible fashion. K562 represents a Tier 1 ENCODE consortium^28^ cell line thus allowing for integration of many genomic datasets generated on these cells. Furthermore, this cell line expresses a large number of vlincRNAs, making it a very attractive system to study these transcripts^24^. K562 cells were transfected with a lentivirus vector pTRE-LwCas13a constructed in this study and containing (1) the gene expressing nuclear-localized Cas13-msfGFP fusion protein under the control of CMV promoter with tetracycline-response elements (TRE) and (2) a constitutively expressed reverse tetracycline transcriptional activator (rtTA) protein under the control of EF1α promoter. Following Dox treatment, we could observe stable 40–50-fold induction of the *Cas13* mRNA expression by RT-qPCR (Fig. 1b). This cell line named TRE-LwCas13a-K562 will be used as the basis for all other experiments below.

### Selection of vlincRNAs and protein-coding mRNA targets for high-throughput screening

We reasoned that exposure to a stress is more likely to reveal biological relevance of transcripts, including lncRNAs. Since K562 is a malignancy-derived cell line, exposing it to anticancer drugs appeared as a rational choice of the survival challenge. The choice of the target transcripts was based on the common assumption that transcripts induced in response to a stress are more likely to represent biologically-relevant components of cellular machinery responsible for coping with that stress. We have chosen 3 anticancer drugs — etoposide (inhibitor of topoisomerase type II)^29^, mirin (inhibitor of the DNA double-strand sensing protein MRE11A)^30^ and imatinib (inhibitor of the *BCR-ABL* oncogene present in K562)^31^ — as the survival challenge treatments for this work. Imatinib has been widely used to treat *BCR-ABL* positive CML in clinic^32^ while etoposide has been used to treat a variety of cancers^29^ including leukemia in certain clinical settings^33^. On the other hand, although mirin is not used clinically, it was found to consistently induce a large number of vlincRNAs in K562 cell line (Cao *et al*., manuscript in preparation).

To identify vlincRNAs involved in an early response to these treatments, we have performed 3 h and 6 h treatments with these drugs and conducted RNA-seq analysis of these samples. As the result, we selected 22 vlincRNAs found to be up-regulated (log_2_ expression fold change >0.58 in both time points) by either imatinib, mirin or etoposide; and 3 vlincRNAs with lower magnitude of up-regulation by at least one of these drugs with log_2_ fold change >0 in both time points for further study (Supplementary Table 2). Eleven of the 22 vlincRNAs were in fact up-regulated by at least 2 of those drugs with log_2_ fold change >0.58 in each time point (Supplementary Table 2). We have tested 12 out of the 22 up-regulated vlincRNAs in a separate biological replicate of the drug treatment experiment by RT-qPCR and observed that 10 were in fact upregulated with log_2_ fold change >0.58 resulting in 83% validation of the RNA-seq results.

We also selected 10 protein-coding genes: *ATM*, *ATR*, *PRKDC*, *MRE11A*, *BCR-ABL*, *EIF4A3*, *ZMAT3*, *AMFR*, *FBXO44*, and *LNPEP*. Since no functional screens have been done under conditions employed in this study, it was not possible to select true positive and negative controls. Still, the first 6 genes have some expectation of biological relevance in our phenotypic assays, while the relevance of the latter 4 under these conditions is not known. *ATM*, *ATR*, and *PRKDC* encode protein kinases representing critical regulators of DNA damage response^34^ and are relevant for this work because of the treatments with etoposide, a drug known to induce DNA breaks^29^. *MRE11A* as mentioned above also functions in the early detection of double-strand DNA breaks and importantly is the known target of another drug used in this study, mirin^30^. *BCR-ABL* oncogene is the major driver of proliferation in K562 cells and also the target of imatinib^35^. *EIF4A3* was shown to be important for K562 viability in a CRISPR/Cas9-based high-throughput screening study under normal growth conditions^36^. On the other hand, gRNAs against 4 other genes *ZMAT3*, *AMFR*, *FBXO44*, and *LNPEP* did not exhibit depletion or enrichment in the same CRISPR/Cas9-based study suggesting that these genes are not important for survival of K562 cells albeit also under normal growth conditions^36^. All 10 genes were expressed in K562, however only *FBXO44* was found to be upregulated by one of the drugs (imatinib) used in this study (Supplementary Table 2).

### High-throughput phenotypic screen

**Figure 2:**
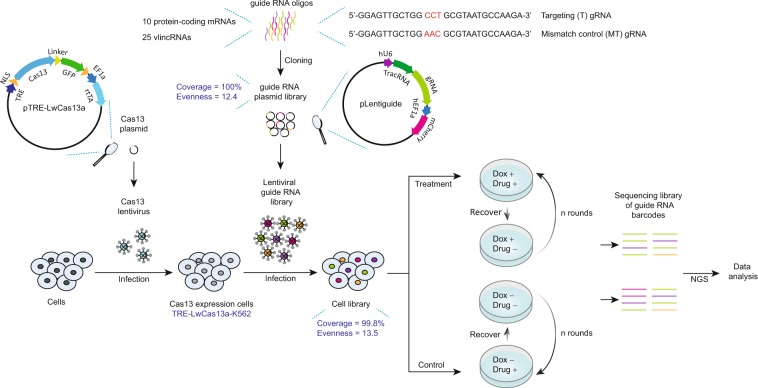
Schematics of the combined survival challenge and the CRISPR/Cas13 high-throughput screening system. Pairs of gRNAs and corresponding mismatch control gRNAs for 10 protein-coding mRNAs and 25 vlincRNAs were designed and cloned into a lentiviral expression backbone to generate a gRNA plasmid library. The latter was then used to generate library of TRE-LwCas13a-K562 cells stably expressing the designed gRNAs. This population of cells was subjected to the survival challenge screen with rounds of drug treatment and recovery (without drug) (5, 4, and 1 rounds for imatinib, mirin and etoposide respectively) in the presence or absence of Dox. Genomic DNA was isolated from the cells at the end of the growth screen to make the sequencing library followed by NGS.

First, for each selected vlincRNA and protein-coding mRNA, we designed respectively 10 and 3–5 pairs of gRNAs (Supplementary Table 3). Each pair of gRNAs consists of a targeting 28-mer gRNA perfectly complementary to the target transcript and the corresponding non-targeting mismatch control gRNA with 3 base mismatches in the positions 12–14 of the 28-mer sequence (Fig. 2, Supplementary Table 3). The gRNA in the CRISPR/Cas13 system was reported to lose most of its targeting ability with 2 central base mismatches^22^. The non-targeting gRNA with 3 base mismatches would thus theoretically serve as an ideal control for the cognate targeting gRNA since the former retains most of its sequence yet should lose most if not all of the targeting function. This feature represents a unique advantage of CRISPR/Cas13 system since it allows for the most precise sequence-specific control for the off-target effects available up-to-date for an RNA-targeting knockdown system — a critical factor in true assignment of a phenotype to the transcript.

We then cloned 294 pairs of gRNAs representing 588 different sequences into a lentiviral vector pLentiguide constructed in this study under the control of a constitutive U6 promoter (Fig. 2). The resulting lentiviral library was then used to transfect the TRE-LwCas13a-K562 cell line at an infection rate of 24% to favor single integration events. One million transfected cells expressing mCherry protein also encoded by the pLentiguide vector under the control of constitutive hEF1α promoter were then selected to form the basis for the library used in the next round of experiments (Fig. 2). Each cell in the library would harbor the Dox-inducible Cas13-msfGFP and a constitutively-expressed gRNA both stably integrated into the genome with the latter representing a unique barcode sequence for that cell.

**Figure 3:**
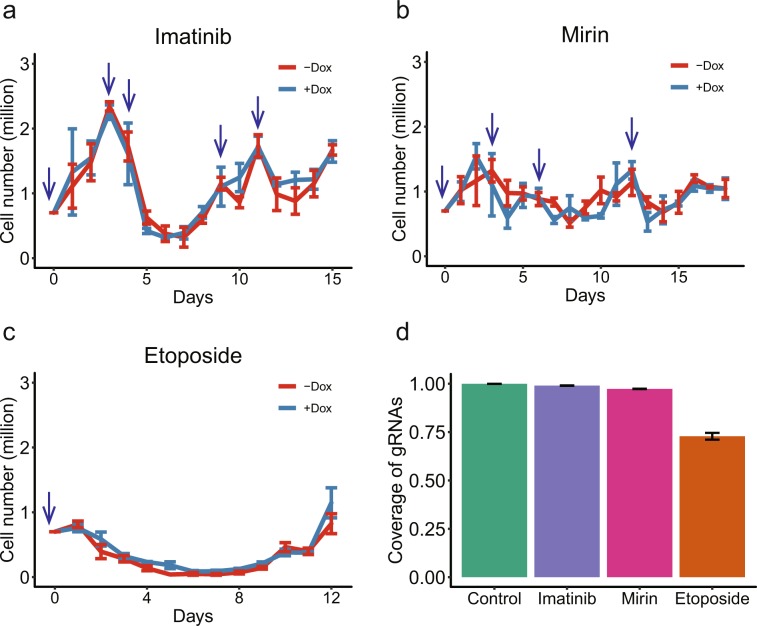
Time course of the survival challenge. (**a**–**c**) Cell numbers (Y-axes) for the different times (X-axes) of imatinib (**a**), mirin (**b**) or etoposide (**c**) treatments in the presence or absence of Dox. The blue arrows indicate drug treatments, error bars show the SE of three biological replicas. (**d**) Coverage of gRNAs observed after each drug treatment, error bars indicate the SE of 12 samples (6 +Dox and 6 −Dox NGS libraries, respectively) or 4 control samples before drug treatment.

As the next step, we applied selective pressure based on a recursive exposure of the library to the drug treatments (Figs. 2 and 3a–c). The goal was to kill cells sensitive to a drug and allow resistant cells to regrow after removing the drug and then repeat the process several times to select cells most resistant to the treatment. After 24 h of the treatment, a drug was washed away and the cells were allowed to recover in culture medium without the drug till the cell shape or doubling rate resembles those of untreated, normally grown K562 cells (see Materials and Methods for more details). Then, the next round of treatment and recovery followed. The concentrations of imatinib (0.75 µM) and etoposide (25 µM) were chosen to be in the range of concentrations detected in the plasma of patients receiving these drugs for chemotherapy^33,37^. As shown in Fig. 3a–c and Supplementary Table 4, the three anticancer drugs had quite different kill/recovery profiles. Imatinib and mirin (75 µM) had relatively moderate effects such that cells could recover within 5 or 6 days after the drug removal (Fig. 3a,b, Supplementary Table 4). In the case of imatinib, sometimes two closely-spaced treatments were required to induce cell death (Fig. 3a, Supplementary Table 4). On the other hand, the etoposide treatment exhibited a much stronger and long-lasting effect with a single treatment resulting in cells dying for at least one week even after removing the drug and taking nearly 2 weeks to recover (Fig. 3c, Supplementary Table 4). Accordingly, 5, 4, and 1 rounds of drug treatment and recovery were performed for imatinib, mirin, and etoposide respectively. Importantly, the etoposide treatment caused the most significant bottle neck with the cell numbers dipping to 9.4% of the original population (6.6 × 10^4^/7 × 10^5^ starting cells) (Fig. 3c, Supplementary Table 4). For comparison, the most significant drops in the populations of the imatinib and mirin treated cells were much less pronounced, represented by the minimum cell numbers of 3.5 × 10^5^ (49.8%) and 6.3 × 10^5^ (90.0%) respectively compared to the 7 × 10^5^ starting cells (Fig. 3a,b, Supplementary Table 4).

The rounds of drug treatment and recovery were done separately in the presence or absence of Dox (Fig. 2). The former should induce Cas13 and as such should also lead to depletion of cells harboring gRNAs against transcripts important for surviving the drug treatment, or on the contrary, increase in cells harboring gRNAs against transcripts that are involved in cell death in response to the drug treatment. At the end of the survival challenge, DNA was isolated from all remaining cells and the gRNA sequences integrated in their genomes were amplified and subjected to next-generation sequencing (NGS). Normalized count of each gRNA in each NGS library would thus represent fraction of cells harboring that gRNA barcode. Each drug treatment was done in parallel on 3 independent batches of cells. Each batch of cells was further subdivided into 2 parts and each was used for NGS library preparation, resulting in 6 replicas for each drug/+Dox and drug/−Dox treatment combination. Frequency of each gRNA — targeting or control — was then measured in each of the 6 replicas of each treatment combination (Supplementary Table 5). Consistent with the most significant bottle neck caused by etoposide, libraries generated from this treatment had the lowest complexity of gRNA sequences with 27% of gRNA sequences being lost from the population compared to 1% and 3% lost in the imatinib and mirin treatments respectively (Fig. 3d).

**Figure 4:**
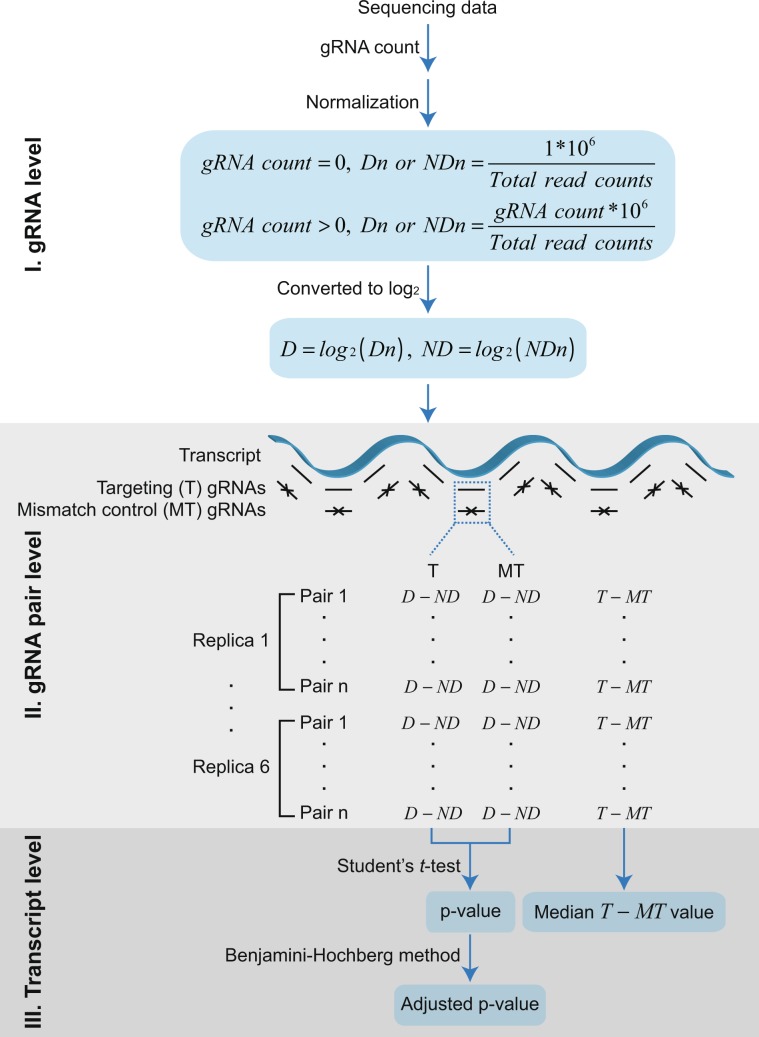
Analysis pipeline of the survival challenge experiments. The analysis can be subdivided into 3 levels — (1) individual gRNA, (2) gRNA targeting-mismatch pair and (3) whole transcript. *Dn* and *NDn* represent normalized gRNA counts in respectively paired +Dox and −Dox samples treated with the same drugs while *D* and *ND* represent the corresponding values in log_2_ space. *T* and *MT* refer correspondingly to the targeting and cognate mismatch control gRNAs.

This study contains four layers of controls to discern the true effect of the target lncRNA depletion: comparison of (1) induced (+Dox) vs un-induced (−Dox) Cas13 and (2) targeting vs non-targeting mismatch gRNA, (3) multiple gRNAs designed against the same transcript and (4) multiple replicas. The true transcript-dependent phenotype should thus be consistently more pronounced in +Dox treatments among all targeting gRNAs compared to the paired mismatch controls in all biological replica. The analytical steps to identify vlincRNAs and protein-coding mRNAs satisfying the above-mentioned criteria are shown in Fig. 4 and described in Materials and Methods. In brief, the analytical step can be subdivided into three levels: (1) individual gRNA, (2) cognate pair of targeting (*T*) and mismatch control (*MT*) gRNAs and (3) set of gRNA pairs targeting each transcript. First, normalized counts for each gRNA in each library were obtained and converted into log_2_ values. Second, for each gRNA, a *D* − *ND* metric was generated by subtracting the log_2_ counts in the −Dox (*ND*) control treatments from the cognate +Dox (*D*) treatment. This metric measured a change in the frequency of that gRNA in response to the +Dox induction of Cas13. Third, for each transcript, a p-value was calculated by performing paired Student’s *t*-test on *D* − *ND* values of the targeting gRNAs vs the mismatch controls from all gRNA pairs designed against that transcript in all libraries from each drug survival experiment. This step identified statistical significance of a Dox-dependent change among all targeting gRNAs for that transcript compared to the mismatch controls. Two levels of statistical significance — “permissive” and “strict” — were used based on p-values respectively un-adjusted or adjusted for multiple testing with a threshold of 0.05 (Materials and Methods). Fourth, for each pair of targeting and mutant gRNAs, we calculated the *T − MT* metric represented by the *D* − *ND* value for the mismatch gRNA subtracted from that of the cognate targeting gRNA. This metric measured the Dox-dependent change in the frequency of the targeting gRNA compared to that of the control. Finally, for each transcript, a median *T − MT* fold change metric was calculated from all gRNA pairs in all libraries from each drug treatment to identify depletion (negative *T − MT*) or enrichment (positive *T − MT*) of that transcript (Materials and Methods).

### Conclusions from the high-throughput phenotypic screen

**Figure 5:**
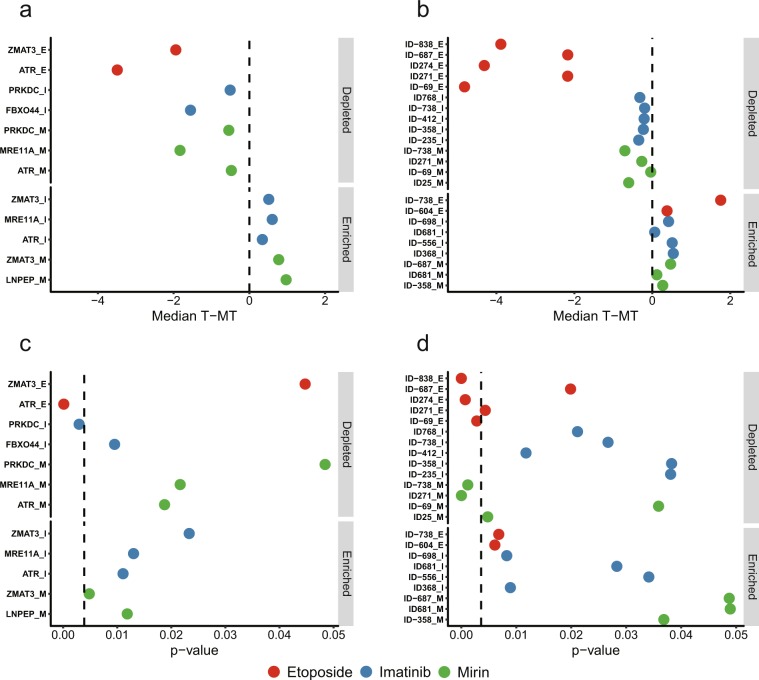
Summary of the positive transcripts identified from the CRISPR/Cas13 survival challenge assays. (**a**–**d**) *T* − *MT* metrics (**a**,**b**) and p-values (**c**,**d**) for the indicated genes and vlincRNAs are shown for the imatinib (I) (blue circles), mirin (M) (green circles) and etoposide (E) (red circles) treatments in which the transcripts were found significant. (**c**,**d**) The dashed lines represent the adjusted p-values of 0.05. The IDs of the vlincRNAs correspond to the Supplementary Table S1 (tab “Non_overlapping_human_vlincRNAs”) of St. Laurent *et al*.^24^ and their coordinates are also listed in Supplementary Table 2 of this study.

Under the permissive criterion, out of the 10 tested protein-coding mRNAs, 6 showed evidence of biological significance. Specifically, targeting gRNAs designed against 5 mRNAs showed statistically-significant depletion in 7 RNA-treatment combinations, while those against 4 mRNAs showed enrichment in 5 combinations (Fig. 5a,c, Supplementary Table 6). Importantly, gRNAs against *ATR* were highly depleted in the etoposide treatment (Fig. 5a,c, Supplementary Table 6). As mentioned above, ATR represents one of the major DNA damage response pathways and has been extensively implicated in response to etoposide^38^. Therefore, it would be expected that knockdown of mRNA encoding this component of DNA damage response would further sensitize cells to the etoposide treatments and cause their removal from the population. Furthermore, *ATR* gRNAs were depleted after the mirin treatments (Fig. 5a,c). Mirin inhibits MRE11A, a component of the double-strand DNA break sensing complex MRN, and as such would also be expected to increase DNA break formation. Interestingly, gRNAs against another kinase involved in double-strand DNA break repair, *PRKDC*, were also depleted in the mirin survival challenge (Fig. 5a,c, Supplementary Table 6). On the other hand, we also observed a signal in 3 out of 4 genes (*ZMAT3*, *FBXO44*, and *LNPEP*) (Fig. 5a,c, Supplementary Table 6) for which previous high-throughput CRISPR/Cas9-based screen failed to find evidence of functionality in K562 cells in the absence of stress challenge^36^. This result suggests that the function of these genes did not reveal itself during normal growth conditions in the CRISPR/Cas9 screen^36^, yet became apparent under the survival challenges used here. Finally, gRNAs against 4 mRNAs (*BCR-ABL*, *EIF4A3*, *ATM*, and *AMFR*) showed no statistically-significant phenotypic effects (Supplementary Table 6).

Importantly, under the same criterion, 16 (64%) out of the 25 vlincRNAs also showed evidence of biological relevance in these assays. Specifically, gRNAs against 11 vlincRNAs were depleted in 14 RNA-treatment combinations, and those against 8 transcripts were enriched in 9 combinations (Fig. 5b,d, Supplementary Table 6). Interestingly, as expected, the etoposide treatment had the most significant effect on vlincRNAs (Fig. 5b,d, Supplementary Table 6). Furthermore, this effect was most significant in depletion — gRNAs against 5 vlincRNAs were depleted with the median *T − MT* of less than −2 (in log_2_ space) relative to the mismatch controls (Fig. 5b, Supplementary Table 6). On the other hand, gRNAs against only 2 vlincRNAs were enriched in this treatment, and none with the *T − MT* of >2 (Fig. 5b, Supplementary Table 6). Etoposide treatment was also highly effective in depleting gRNAs against mRNAs — the corresponding *T − MT* for *ATR* and *ZMAT3* were −3.5 and −1.9 (Fig. 5a, Supplementary Table 6) with none of the gRNA sets enriched by this treatment. Interestingly, *ZMAT3* has been previously implicated in DNA damage response^39^ potentially explaining its importance in responding to the etoposide treatment. On the other hand, the mirin and imatinib treatments were much milder in terms of the effects on gRNAs for both vlincRNAs and mRNAs (Fig. 5a,b, Supplementary Table 6). The only exceptions were *MRE11A* in the mirin treatment and *FBXO44* in imatinib, with the corresponding *T − MT* of −1.8 and −1.6 (Fig. 5a, Supplementary Table 6). The effect of *MRE11A* knockdown could be explained by a synergism with inhibition of the product of this mRNA by mirin. In this respect, the imatinib treatment upregulated *FBXO44* mRNA (Supplementary Table 2), consistent with its potential involvement in cellular response to this drug.

Using the strict criterion — adjusted p-value < 0.05 — we found 2 genes and 5 vlincRNAs significant (Fig. 5c,d, Supplementary Table 6). While not passing the threshold, the adjusted p-values for the remaining 4 genes and 7 vlincRNAs were in a marginally significant range (0.05–0.1) (Supplementary Table 6). The 2 genes, *ATR* and *PRKDC* were found to be depleted under the etoposide and imatinib survival challenges respectively (Fig. 5a,c, Supplementary Table 6). While as mentioned above ATR activity is known to be involved in the etoposide response^38^, the relationship between PRKDC and imatinib is less clear. Still, two lines of published evidence provide potential connection between the two. First, BCR-ABL regulates PRKDC level^40^ and second, imatinib and PRKDC inhibitors can have synergistic effects on leukemia cells^41^. Furthermore, 3 vlincRNAs were found to be depleted under the etoposide challenge and 2 — under the mirin one (Fig. 5d, Supplementary Table 6). As expected, the fold depletion under the etoposide treatments were much greater for all transcripts than under other treatments with the corresponding of *T − MT* metrics ranging from −4.8 to −3.5 (etoposide) and −0.7 to −0.3 (other drugs) (Fig. 5a,b, Supplementary Table 6).

**Figure 6:**
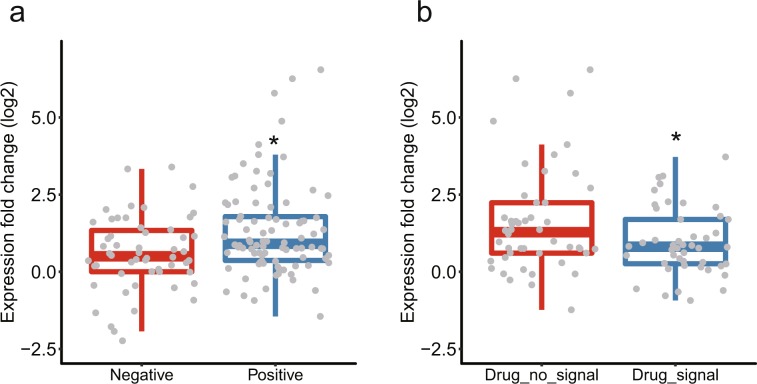
Relationship between the expression fold changes and the behavior in the phenotypic screen for the vlincRNAs. (**a**) Box plots for the log_2_ of expression fold changes (Y-axis) for all 3 drug treatments and in both 3 h and 6 h time points (Supplementary Table 2) for vlincRNAs that do (right) and do not (left) yield statistically significant signal under the permissive criteria in the phenotypic screens. (**b**) Box plots for the log_2_ of expression fold changes (Y-axis) for the vlincRNAs positive in the phenotypic screens and in drug treatments where the phenotypes could (right) and could not (left) be detected. Asterisks indicate significant differences under Student’s *t*-test (p-value < 0.05, see text for more details).

Interestingly, we found that a survival challenge drug treatment where a vlincRNA was positive sometimes differed from the one that induced the transcript in the expression assays (Fig. 5b, Supplementary Table 2). To further explore this phenomenon, we performed a deeper analysis of the expression fold changes detected by the RNA-seq data by comparing them with the results of the phenotypic screens. First, we compared the expression fold changes caused by the treatments with all 3 drugs for vlincRNAs positive in the phenotypic assays with those for the negative vlincRNAs under the permissive threshold. Interestingly, we found that in general, the fold changes of the former were higher than the latter (respective median log_2_ fold change 0.86 vs 0.50, p-value 0.0018, Student’s *t*-test, Fig. 6a). This suggests that vlincRNAs found to be functional in the phenotypic screens are also induced more in response to stresses used in these screens. However, when we compared the expression fold changes for only the positive vlincRNAs and in the drug treatments that caused the phenotypes vs the ones that did not, we found, surprisingly, that the fold changes had a statistically-significant tendency to be higher in the latter (respective median log_2_ fold change 0.81 vs 1.28, p-value 0.016, Student’s *t*-test, Fig. 6b). In other words, vlincRNAs positive in the phenotypic screens with certain drug treatments were indeed induced by those drugs in the expression assays (Fig. 6a). However, those vlincRNAs were induced even more by the drugs where no effect could be observed in the phenotypic screens (Fig. 6b). Based on these observations, we would like to propose the following explanation that connects expression changes of transcripts in response to the drugs with the phenotypic consequences of their knockdowns. On one hand, stronger induction at the expression level identifies transcripts more relevant to a cellular response to a particular stress. On the other hand, strong induction also increases the abundance of the transcript making the knockdown to a phenotypically-relevant level more challenging. Therefore, it appears that our ability to obtain a phenotype balances between functionality of a transcript and by how much its abundance exceeds the threshold required to cause the phenotype.

## Discussion

For the first time, in this study we have (1) shown that CRISPR/Cas13 system can be used in a high-throughput phenotypic assay setting and (2) provided a proof of functionality for a novel class of lncRNAs based on the application of this system. While a multitude of studies have provided evidence supporting functionality of lncRNAs, as mentioned above, currently used reverse-genetics methods suffer from major technical and interpretational issues that often preclude unequivocal assignment of biological relevance to the assayed transcripts using these techniques. In this regard, the CRISPR/Cas13 system has some key advantages — most notably, the high level of specificity and the ability to use closely related mismatch controls — over the other methods used to study lncRNAs.

Furthermore, we observed similar fractions — up to 60% vs 64% of functionally significant transcripts among the tested protein-coding mRNAs and vlincRNAs. Moreover, the magnitude of functional relevance (as measured by the *T − MT* metric) correlated most strongly with the strength of selection rather than with the type of transcripts. The most significant phenotypic effect was achieved in the etoposide survival challenge that also caused the highest cellular mortality for both vlincRNAs and genes. Interestingly, for both types of transcripts, the depletion was more common than the enrichment, suggesting that these transcripts are required for survival rather than for limiting cell growth or cell death under the drug treatments. In this respect, our results are consistent with the CRISPR/Cas9 high-throughput screen in K562 and other cell lines where the depletion of gRNAs was also more common than the enrichment^36^.

The fairly high fraction of functional vlincRNAs observed in this study could be explained by several factors. First, the choice of the cell line that expresses the target vlincRNAs at a relatively high level^24^ and thus potentially indicating their functionality in this cell type. Second, we selected vlincRNA induced by the stress conditions (and thus likely involved in cellular responses to those) for the phenotypic assays based on survival challenges employing the same stresses. Third, as mentioned above, we found that a proper choice of conditions under which the phenotypes are assayed is quite important for uncovering biological relevance of the targeted transcripts. Here, we used a survival challenge approach where cells are subjected to recurrent rounds of stress and recovery. Not surprisingly, we found that the treatment inducing the strongest selection pressure also provided the strongest support for the biological significance of vlincRNAs and mRNAs. All this illustrates the importance of carefully selecting the experimental system and the target lncRNAs for functional characterization.

The survival challenge in combination with CRISPR/Cas13 strategy used here can be applied on a larger scale to uncover transcripts — both coding and non-coding — that could be involved in mediating resistance or sensitivity to anticancer drugs. This is exemplified by the identification of known interactions such as *ATR*-etoposide or *MRE11A*-mirin as well as some potentially novel one such as *FBXO44*-imatinib, *PRKDC*-imatinib, *ZMAT3*-etoposide and others represented by the vlincRNAs. While the CRISPR/Cas9-based approaches have been employed in screens for genes involved in drug resistance^42^, they cannot readily attribute the phenotypes to the transcripts. The approach described here does not have this limitation. On the other hand, like all other knockdown methods, it can not achieve complete depletion of the target transcript and as our work shows, likely fails to detect phenotypes of transcripts whose abundance after depletion does not fall below the phenotypic threshold. Our inability to detect a phenotypic signal for such highly expressed genes as *EIF4A3* could likely be explained by this factor. However, the ability of this technique to target RNA and its superior specificity should make CRISPR/Cas13 a mainstream reverse-genetics methodology in the field of lncRNA research.

## Conclusions

LncRNAs represent an exciting class of transcripts whose biological significance remain highly controversial. Recent studies have brought artifacts associated with the long-known flaws in the commonly used RNA knockdown techniques to the fore. Combined with the recent failures to obtain obvious *in vivo* phenotypes for multiple lncRNAs, these issues challenge the validity of the massive amount of the previous data supporting the functionality of this class of transcripts generated using these techniques. As such, a clear need exists for development of novel methods for reverse-genetics analysis of lncRNAs free of the known issues plaguing the existing techniques or at least having superior performance than the latter.

Here, we show the feasibility of application of one such promising technique based on the CRISPR/Cas13 system by using it to address biological relevance of a large fraction of the tested lncRNAs belonging to the vlinc class. We provide an example of application of this technique in a context of a stable expression system that could be subjected to prolonged survival challenges. This study can serve as a general guideline for other such studies aimed at investigating biological functionality of lncRNAs and mRNAs in anticancer drug response or potentially, in any other stresses that result in cell lethality or reduced growth or survival. Finally, we believe that this approach can be scaled up to the whole genome level.

## Materials and Methods

### Plasmids and cell lines

The pC034-LwCas13a-msfGFP-2A-Blast plasmid expressing LwCas13a protein stabilized by fusion with msfGFP (LwCas13a-msfGFP) and flanked by N-terminal and C-terminal nuclear localization sequence (NLS)^22^ was a gift from Feng Zhang (Addgene plasmid #91924; http://n2t.net/addgene:91924; RRID: Addgene_91924). It was further modified by substitution of the EF1α core promoter of LwCas13a with a CMV promoter containing 7 TRE’s, as well as the insertion of rtTA driven by EF1α core promoter to create pTRE-LwCas13a. The gRNA expression cassette including the U6 promoter was obtained from pC016-LwCas13a^22^ (a gift from Feng Zhang (Addgene plasmid #91906; http://n2t.net/addgene:91906; RRID: Addgene_91906)). It was further modified by replacement of two BbsI recognition sites with a *ccdB* cassette flanked by two BsaI recognition sites, insertion of mCherry selection marker driven by hEF1α promoter, and assembled in a lentiviral expression backbone to generate the lentiviral vector pLentiguide (Fig. 2). The vectors were generated by SyngenTech (Beijing, China) and confirmed by Sanger sequencing.

Human CML cell line K562 was obtained from Cell Bank of Chinese Academy of Sciences. The K562 cell line expressing Dox-inducible Cas13-GFP fusion (TRE-LwCas13a-K562) was generated by SyngenTech (Beijing, China) by transfection with lentivirus generated from the pTRE-LwCas13a plasmid in the 293FT packing cell line. All K562 cell lines were maintained in RPMI 1640 medium (ThermoFisher Scientific, US) supplemented with 10% fetal bovine serum (ThermoFisher Scientific, US) and 1% pen/strep (ThermoFisher Scientific, US) at 37 °C in 5% CO_2_.

### Drug treatments for expression analysis and RNA-seq

For the expression analysis to identify transcripts induced by specific drugs, K562 cells (5 × 10^5^ cells/ml) were grown in RPMI 1640 (ThermoFisher Scientific, US) supplemented with 10% fetal bovine serum (ThermoFisher Scientific, US) in a 6-well plate for 16 h and treated with either an anticancer drug (1 μM imatinib, 100 μM mirin, or 100 μM etoposide) or DMSO as a control for 3 h or 6 h. After the treatment, RNA was isolated with E.Z.N.A. Total RNA Kit I (Omega) and RNA-seq was performed on Illumina platform (Hiseq X Ten) by Novogene Corporation (Beijing) using rRNA-depletion protocol and paired-end 150 bp strategy on a 10 GB scale.

Expression levels of genes were estimated based on the RNA-seq data using Salmon software^43^ for the reference human transcriptome (hg38) from the Ensembl database^44^ and 2,721 vlincRNA transcripts taken from the previous publications^24,45^. The raw read counts were normalized using DESeq. 2 package^46^ in *R* environment. The expression fold change (FC) of gene *i* induced by drug *j* was calculated as:

$$F{C}_{i,j}=lo{g}_{2}[(NR{C}_{i,j}+1)/(NR{C}_{i,DMSO}+1)]$$

where *NRC*_*i*, *j*_ and *NRC*_*i*, *DMSO*_ are the normalized read counts of gene *i* in drug-treated and DMSO-treated samples respectively. To validate the RNA-seq results, RT-qPCR was performed on 30 ng or 60 ng of cDNA from RNA derived from a separate biological replicate of the drug treatments as previously described^27^.

### Depletion of gene and vlincRNAs with CRISPR/Cas13 using transient transfection assays

Pairs of sense and antisense oligonucleotides for each gRNA were annealed in 50 μl volume containing 10 mM Tris, pH 7.5–8.0, 50 mM NaCl and 2 μM of each oligo as follows: after incubating at 95 °C for 5 min, the temperature was gradually decreased at 1 °C/min to 25 °C. The annealed products were then cloned into BbsI-digested pC016-LwCas13a vector. Plasmids were isolated by PureLink^TM^ HiPure Plasmid Filter Midiprep Kit (Invitrogen) following the manufacturer’s instructions.

Electroporation was done with Neon® Transfection System (Invitrogen) using the 100 µl kit following the manufacturer’s instructions. Briefly, K562 cells were grown to 70–90% confluency, harvested and washed in phosphate buffered saline (PBS) without Ca^2+^ and Mg^2+^. Then, 1 × 10^6^ cells were resuspended in the Resuspension Buffer R containing 3.75 µg pC034-LwCas13a-msfGFP-2A-Blast and 6.25 µg pC016-LwCas13a guide expression plasmid, brought to 100 µl final volume and subjected to electroporation. Afterwards, the cells were transferred immediately to a 12-well plate containing 1 ml RPMI 1640 (ThermoFisher Scientific, US) with 10% fetal bovine serum (ThermoFisher Scientific, US) prewarmed to 37 °C, and incubated for 24 h prior to RNA isolation with E.Z.N.A. Total RNA Kit I (Omega). Depletion of the targeting gRNAs relative to the Gluc gRNA control was evaluated by RT-qPCR. List of gRNA sequence and RT-qPCR primers can be found in Supplementary Table 1.

### Design of gRNA library

For each vlincRNA and protein-coding mRNA, respectively 10 and 3–5 pairs of targeting and mismatch control gRNAs were designed. First, targeting gRNAs were selected under the following criteria: (1) repetitive sequences as defined by the RepeatMasker were excluded; (2) gRNA sequence had to have average uniqueness as defined by the “Uniqueness of 20 bp Windows from ENCODE/OpenChrom (Duke)” track of the UCSC Genome Browser^47^ >0.7; (3) gRNAs had to contain 40–60% GC, (4) no homopolymeric stretches >3 bases and (5) no dinucleotide runs >2 pairs; (6) for protein-coding genes, gRNAs were designed against exons only. Second, the corresponding mismatch control gRNAs were designed by changing bases 12–14 (first base of the 28-mer being 1) of the targeting gRNA sequences to create mismatching sequence. Finally, the sequences required to anneal to the cloning sites, AAAC and AAAA, were added to the 5’ end of gRNA and reverse complement sequence respectively. To target *BCR-ABL*, exons 1–14 of *BCR* (UCSC ID uc002zww.3) were used for the gRNA design. For *MRE11*, *ZMAT3*, and *FBXO44*, only three pairs of gRNAs could be selected using these criteria. In total, 588 gRNA sequences were designed (Supplementary Table 3).

### Construction of lentiviral gRNA plasmid library

Pairs of oligonucleotides corresponding to the sense and antisense gRNA sequences were annealed as above and mixed together to generate a pool with equal amount of each annealed oligonucleotide. One microgram of the pLentiguide vector was digested in 25 μl of 1× CutSmart buffer and 30 U BsaI-HFv2 (New England Biolabs) at 37 °C for 12 h, followed by 20 min incubation at 65 °C to inactivate the enzyme. Pooled annealed oligonucleotides (73 ng) were ligated with ~150 ng of the digested pLentiguide vector in 20 μl using 600 U of T4 DNA ligase (New England Biolabs) at 16 °C for 9 h, followed by enzyme inactivation at 65 °C for 10 min. Twenty ligation reactions were performed in parallel.

Stbl2 *E*. *coli* competent cells (100 μl) (Shanghai Weidi Biotechnology) were thawed on ice, gently mixed with 10 μl of either the ligation mix or sterilized H_2_O as a negative control and incubated on ice for 15 min, heat-shocked at 42 °C for 45 s in a water bath and immediately placed on ice for 5 min. LB medium (450 μl) was added directly into the solution and incubated at 220 rpm (37 °C) for 1 h. Nineteen transformations with each of the above mentioned ligation reactions were performed. The bacterial cells were spread onto 38 15 mm LB agar plates in the presence of 100 μg/ml ampicillin (275 μl/plate), and incubated at 37 °C for 16 h. Altogether 36,791 bacterial colonies (coverage >60×) were obtained. Ten colonies from different plates were randomly picked for Sanger sequencing. To preserve the diversity of the library, for the lentiviral vector production, *E*. *coli* colonies were scraped from the plates after transformation and used directly for plasmid isolation with PureLink^TM^ HiPure Plasmid Filter Midiprep Kit (Invitrogen) following the manufacturer’s instructions.

### Assessing plasmid library coverage and evenness by NGS

The plasmid DNA (10 ng) was resuspended in 20 μl first round PCR solution (1× Taq buffer, 0.4 μl of 2.5 mM dNTP mix (Takara), 1 U of Taq DNA polymerase (Tiangen) and 0.5 μM of each of the following primers P5LentiG-FW (CTACACGACGCTCTTCCGATCTACGAAACACCGGATTTAGACTAC) and P7LentiG-RV (CAGACGTGTGCTCTTCCGATCTGGGCACCGGAGCCAAGCTTAA) and subjected to a 2-step PCR. In the first step, initial denaturation at 94 °C for 3 min was followed by 6 cycles of denaturation at 94 °C for 30 s, annealing at 55 °C for 30 s, and extension at 72 °C for 1 min, as well as a final extension at 72 °C for 10 min. For second round of amplification, 2 μl of the first round PCR products were used as the template, and amplified with Illumina-P5 primer (AATGATACGGCGACCACCGAGATCTACACTCTTTCCCTACACGACGCTCTTCCGATCT) and Illumina-P7 primer (CAAGCAGAAGACGGCATACGAGATCGTGATGTGACTGGAGTTCAGACGTGTGCTCTTCCGATCT) for 15 cycles using the same conditions as in the 1^st^ step. After purification with 1 volume of VAHTS DNA Clean Beads (Vazyme), the DNA was dissolved in 21 μl H_2_O, the concentration was measured by Qubit 3.0 fluorometer using Equalbit^TM^ dsDNA HS Assay Kit (Vazyme). NGS was performed on Illumina platform (Hiseq X Ten) using paired-end 150 bp strategy by Novogene Corporation (Beijing) and 1 GB of raw data was collected. The following parameters were calculated: (1) coverage —  the fraction of gRNAs detected compared to the total gRNAs (588) and (2) after sorting gRNAs by the counts, evenness was calculated as 90^th^ %-ile gRNA count/10^th^ %-ile gRNA count. Our plasmid library had the coverage of 100% and evenness of 12.4 (Fig. 2, Supplementary Table 7).

### Construction of gRNA cell library

Lentivirus particles were produced by transfecting the 293FT packaging cell line with the gRNA plasmid library and used to transfect the TRE-LwCas13a-K562 cell line expressing Dox-inducible Cas13 at an infection rate of 24%. One million transfected cells containing the gRNA sequences (TRE-LwCas13a-gRNA-K562) were selected by flow cytometry (BD CytoFLEX) using mCherry as the selection marker and expanded.

For evaluating the coverage and evenness of the gRNAs in the initial TRE-LwCas13a-gRNA-K562 cell library used for all subsequent experiments, DNA was extracted from 2 × 10^6^ cells by TIANamp Genomic DNA Kit (Tiangen) following manufacturer’s instructions and dissolved in 50 μl H_2_O. The DNA (1 μg) was mixed with 2.5 U Taq DNA polymerase (Tiangen), 1 μl of 2.5 mM dNTP mix (Takara), 2.5 μl of 10 μM P5LentiG-FW and P7LentiG-RV primers, and 1× Taq buffer in a final volume of 50 μl. The PCR conditions were as above, except that 10 cycles were used in the first round. The PCR products were purified with 1 volume of VAHTS DNA Clean Beads (Vazyme) and dissolved in 21 μl H_2_O. To maintain a theoretical average of 1000× (cells/gRNA) coverage of the gRNA library, for each library, 5 PCR reactions with a total of 5 μg DNA template were performed and pooled together. The PCR products were sequenced on the Illumina platform as above, and the library coverage and evenness were evaluated with the same rules used for the lentiviral gRNA plasmid library. The cell library had the coverage of 99.8% and the evenness of 13.5 (Fig. 2, Supplementary Table 7).

### Survival challenge assays

For the drug treatments, the TRE-LwCas13a-gRNA-K562 cells were plated into 10 ml medium supplemented with 1 μg/ml Dox (Macklin Inc, 24390-14-5) in a T25 flask (7 × 10^4^ cells/ml) and incubated for 3 days, then 7 × 10^5^ cells (~1000× cells/gRNA coverage) were seeded into each well of a 6-well plate with 2 ml medium containing 1 μg/ml Dox and either one of the anticancer drugs (0.75 μM imatinib (AbMole BioScience, USA), 75 μM mirin (AbMole BioScience, USA), or 25 μM etoposide (AbMole BioScience, USA). In parallel, −Dox control cells were treated with water instead of Dox and the same doses of the drugs. After 24 h, cells were collected, washed twice with 1 ml RPMI 1640 to remove the drugs and resuspended in 2 ml fresh medium with 1 μg/ml Dox or water for recovery. For each well, cells were passaged daily with the maximum density of 3.5 × 10^5^ cells/ml, also 45 μl of cells were collected and mixed with 45 μl of medium and 10 μl of trypan blue staining solution (0.4%, Solarbio) to evaluate the fraction of the live cells. The next round of drug treatment was performed when most cells recovered the normal shape or the doubling rate of the untreated cells (Fig. 2, Supplemental Table 4). Three independent biological replicates were performed for each drug/+Dox or drug/−Dox combination. At the end of the survival challenge, cells from each biological replicate were split into two parts and genomic DNA was harvested using TIANamp Genomic DNA Kit (Tiangen) from each.

### Analysis of gRNA profiles after the survival challenges

Two rounds of PCR were performed to prepare the sequencing library. For the first round of PCR, 1 μg of the genomic DNA was mixed with 50 μl PCR solution (1× Taq buffer, 2.5 U Taq DNA polymerase (Tiangen), 1 μl of 2.5 mM dNTP mix (Takara), and 2.5 μl of 10 μM P5LentiG-FW and P7LentiG-RV primers). PCR conditions were as follows: initial denaturation at 94 °C for 5 min; 10 cycles of denaturation at 94 °C for 30 s, annealing at 55 °C for 30 s and extension at 72 °C for 1 min; further extension at 72 °C for another 10 min. For the second round of PCR, 2 μl products from the 1^st^ round PCR were mixed with 1 U of Taq DNA polymerase (Tiangen), 0.4 μl of 2.5 mM dNTP mix (Takara), 1 μl of 10 μM Illumina-P5 and Illumina-P7 primers, and 1× Taq buffer to a final volume of 20 μl. The PCR conditions were same as the 1^st^ round PCR except for 20 cycles of amplification. Five parallel PCR reactions were performed for each aliquot of cells to generate each library. The 5 PCR reactions were then pooled and purified with 1 volume of VAHTS DNA Clean Beads (Vazyme) and subjected to NGS as described above.

The analytical steps used to identify transcripts depleted or enriched in response to the survival challenges based on changes in their gRNA distributions are described in the main text and shown in Fig. 4. For each gRNA, number of raw reads containing its exact sequence were counted in each library. The count was then normalized to the total count of all gRNAs in the library with zero count values converted to 1 before normalization. The normalized counts were then converted to log_2_ values. Statistical significance of depletion or enrichment for each transcript was calculated between paired *D* − *ND* values for all target (*T*) and mismatch control (*MT*) gRNAs among all 6 replicas using one-sided Student’s paired *t*-test. Thus, for a vlincRNA with 10 gRNAs, a comparison of 60 (*T*) vs 60 (*MT*) *D* − *ND* values was conducted. The p-value was also adjusted for multiple comparisons with the Benjamini-Hochberg method in *R* environment. A p-value of 0.05 (either raw or adjusted) was chosen as a permissive or strict threshold to identify the positive transcripts as shown in Fig. 5.

## Supplementary information

Supplementary Tables.

## Data Availability

Processed data used to make conclusions in the text are presented in Supplementary Tables and referred to in the appropriate places in the main text, figure legends and Materials and Methods section. The NGS data will be deposited in a public archive upon acceptance.
